# The transtheoretical model is an effective weight management intervention: a randomized controlled trial

**DOI:** 10.1186/s12889-020-08796-1

**Published:** 2020-05-11

**Authors:** Patrícia Pinheiro de Freitas, Mariana Carvalho de Menezes, Luana Caroline dos Santos, Adriano Marçal Pimenta, Adaliene Versiani Matos Ferreira, Aline Cristine Souza Lopes

**Affiliations:** 1 Research Group in Nutrition Interventions, Universidade Federal de Minas Gerais, Belo Horizonte, Minas Gerais Brazil; 2 Department of Nutrition, School of Nursing, Universidade Federal de Minas Gerais, Belo Horizonte, Minas Gerais Brazil; 3 Department of Maternal and Child Nursing and Public Health, School of Nursing, Universidade Federal de Minas Gerais, Belo Horizonte, Minas Gerais Brazil

**Keywords:** Obesity, Intervention studies, Feeding behavior, Theoretical models, Primary health care

## Abstract

**Background:**

Given the current worldwide epidemic of obesity**,** there is a demand for interventions with higher impact, such as those carried out in the primary health care (PHC) setting. Here we evaluate the effect of intervention performed according to the stages of change of the transtheoretical model (TTM) for weight management.

**Methods:**

This randomized controlled trial in Brazilian PHC offered free physical exercise and nutrition education. The participants were women, aged 20 years or older who were obese or overweight, users in PHC service. The intervention group (IG, *n* = 51) received the same orientation as the comparison group (CG, *n* = 35) plus individual health counseling based on the TTM aimed at weight loss, which lasted 6 months. The outcome measures were anthropometric, food, and nutrient profiles. Inflammatory parameters were evaluated in a random subsample. The inter-group and intra-group differences were evaluated using interntion-to-treat analysis, and analysis of covariance (ANCOVA) used to assess intervention effectiveness.

**Results:**

There was a difference between groups of − 1.4 kg (CI95%: − 2.5; − 0.3) in body weight after the intervention. About 97% of women in the IG reported benefits of the intervention and presented positive changes in diet, biochemical markers, and anthropometry. The IG showed better body mass index, resistine, and blood glucose results compared to the CG during follow-up.

**Conclusion:**

The individualized TTM-based intervention, combined with usual care, was an effective strategy in PHC. These results should encourage the use of interdisciplinary practices; nevertheless, research to identify additional strategies is needed to address barriers to weight maintenance among obese low-income women.

**Trial registration:**

The trial is registered with Brazilian clinical trials under the code: RBR-8t7ssv, Registration date: 12/12/2017 (retrospectively registered).

## Background

Obesity and overweight are major global public health problems. In 2016, more than 1.9 billion adults in the world were overweight, and 13% were obese [1]. These conditions increase individuals’ risk of non-communicable diseases (NCD), affect their quality of life, and reduce life expectancy [2].

Weight loss can restore health and wellbeing [2]. Thus, effective and sustainable strategies should be performed to treat obesity. Considering the complex multifactorial etiology of obesity, treatment can involve changes in lifestyle, psychological strategies, pharmacologic treatment, and bariatric surgery [3]. Nevertheless, higher-impact interventions involving the community or most of the overweight population, such as those carried out in the primary health care (PHC) setting, are needed [4].

Several strategies have been used to treat obesity, and these typically emphasize physical activity and dietary behaviors [4], including restricting portion sizes and calories, reducing carbohydrate or fat intake, increasing fruit and vegetable intake, and increasing daily physical activity and planned exercise [3, 5, 6].

Counseling for weight loss that focuses on physical activity and diet has been shown to help reduce body weight, but with modest effects [5]. However, considering behavior as the key to weight loss, behavior-change theories have been proposed as important tools to help participants to achieve healthier behaviors and to deal with their obstacles, thus changing attitudes and contributing to sustainable weight loss [3, 6–8]. Theories that identify the individual’s readiness to change might be useful, since the majority of people are not ready to change their behavior and, therefore, will not be able to follow traditional action-oriented diet programs [7, 9]. The transtheoretical model (TTM) assumes that behavioral change is complex, unfolding in a sequence of stages (Table 1) [7, 9]. The stages of change (SOC) describe the individual’s current intention and engagement toward a targeted health-related behavior [7, 9]. In the first stage, called pre-contemplation, the individual has no intention to change behavior. The second stage is contemplation, when the individual is aware that a problem exists, but has not yet made the commitment to take action. At the preparation stage, people have the intention to take action in the next month. At the action stage, people begin to modify their behavior, and at the last stage (maintenance), the new behavior has continued for at least 6 months [7, 9]. In addition to the SOC, the TTM is based on three other dimensions: (i) processes of change; (ii) decisional balance; and (iii) self-efficacy. Processes of change are activities that individuals engage in when they attempt to modify problematic behaviors. The decisional balance is the comparative potential gains and losses to change, and self-efficacy is the degree of confidence individuals have in maintaining their desired behavior change in situations that often trigger a relapse. Decisional balance and self-efficacy vary depending on the stage of change [7, 9]. Actions based on these dimensions can help to understand the behavioral change and, therefore, are used to assess the patients’ motivation and intervention design [7, 10].

**Table 1:** Stage of changes and processes of change used in the intervention group

Stage of Change	Who are they?	Processes of change used in the intervention
Pre-contemplation stage	Individuals that do not intend to change their behavior in the foreseeable future	• Consciousness raising
		• Dramatic relief (emotional arousal)
		• Reassessment of Environment
Contemplation stage	Individuals that recognize the need for change but action is required to shape their motivation	• Consciousness raising
		• Dramatic relief (emotional arousal)
		• Reassessment of Environment
		• Self-reevaluation
Preparation stage	Individuals that are ready to change their behavior within 30 days	• Self-reevaluation
		• Social liberation
		• Self-liberation
Action stage	Individuals that are capable of short and immediate changes for a period of up to six months	• Self-liberation
		• Contingency management
		• Social liberation
		• Conterconditioning
		• Stimulus control
Maintenance stage	Individuals’ behavior has been changed over six months, requiring now the prevention of relapse and consolidation of gains	• Contingency management
		• Social liberation
		• Conterconditioning
		• Stimuus controll

Although TTM is widely used to treat various health behaviors, there is little evidence of its effectiveness to induce weight loss [9, 11]. Most evidence is produced in high-income countries and shows a reduction of weight, body mass index, and percentage of body fat [7, 12], in addition to an increase in physical activity and an improvement in cholesterol levels and glycemia [5, 13]. TTM-based weight loss interventions use multiple behaviors that exhibit greater compliance with bodyweight control, rather than using a single behavioral approach [8]. Among the most evaluated behaviors are portions control, dietary fat, fruit and vegetable intake, and the practice of physical activity.

Considering the positive results already observed through the TTM for weight loss and the lack of interventions to weight loss in the PHC context, this study aimed to evaluate the effect of an individualized intervention based on the TTM for the weight management of overweight and obese women in a Brazilian PHC service. The hypothesis of this study is that interventions based on behavioral theories, such as TTM, are more effective for weight loss than the typical PHC.

## Methods

### Study design and setting

This is a randomized controlled trial comparing two groups, the intervention group (IG) and the usual care comparison group (CG). Eligible participants were recruited from “Programa Academia da Saúde” (PAS) between March to August 2012.

The public health system in Brazil has invested heavily in primary care. The system is divided into different health services, such as basic health units and the PAS. This program aims to enhance population autonomy through actions that promote health in socially deprived areas [14]. The PAS service offered, at no cost, physical exercise, and food and nutrition education actions conducted by health professionals, for the entire population. More than 2900 Brazilian municipalities now have a PAS unit. The participants have a vulnerable situation, with low socioeconomic condition and high prevalence of NCD [15]. The innovative PAS service represents an important initiative to promote health and prevent and control the prevalence of NCD, especially in populations with high social vulnerability. The program is an important service of the Health Care Networks, including care of NCD such as obesity, functioning as a gateway to the public health system.

The study was carried out in a PAS unit implemented in 2008. All participants from the PAS unit analyzed were included in the study if they met the following inclusion criteria: female (the majority of users in PAS, making it possible to obtain sufficient sample for the trial) [15], have participated in service activities in the previous month (which enabled the nutritional evaluation for sample selection), aged 20 years or older, and obese adult (individuals aged 20 to 59 years and body mass index - BMI ≥ 30 kg/m^2^) [16] or overweight elderly (individuals aged 60 years or over and BMI ≥ 27 kg/m^2^) [17]. The exclusion criteria included individuals with cognitive difficulties, which made it impossible to answer the interview, or who were pregnant because they present specific recommendations for weight control.

**Fig. 1:**
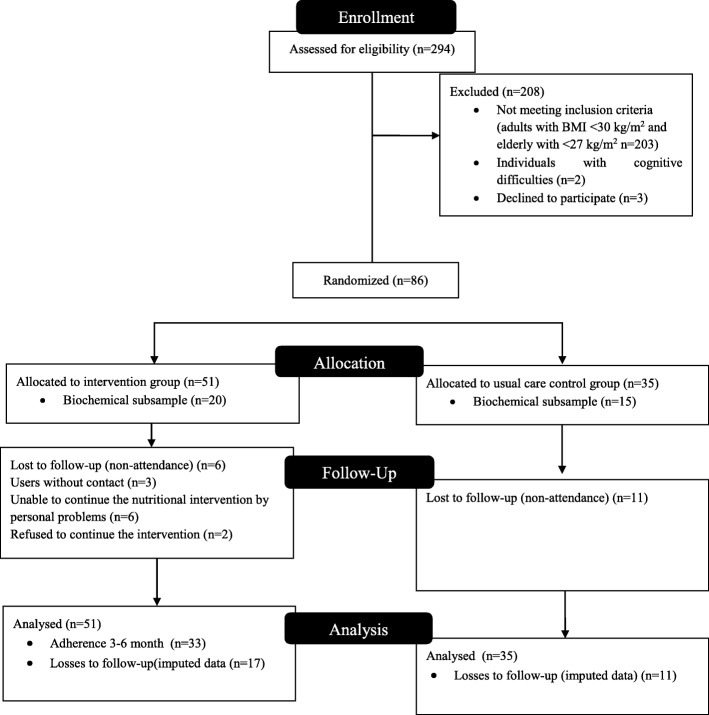
Participant flow

The sample of this study included all individuals from the PAS unit who met the inclusion and exclusion criteria. Of 294 individuals who were screened, 89 were eligible, and 86 consented to participate (Fig. 1). Biochemical profile analyses were performed on a subsample (40%) calculated based on an average of other experimental designs, and the participants were randomly selected [17]. The considered sample size effect allows 80% of the power to achieve 5% weight reduction, as reported in the literature [18], and possible attrition of 30%. A posteriori sample calculation was performed, considering a tolerable relative error of 5%, and 95% confidence interval, obtaining an average sample size estimated at 39 participants.

### Randomization and blinding

A blinded researcher performed the participant allocation for CG and IG through a random numbers table. To maximize the proportion of participants receiving the intervention and the statistical power available to compare the groups, the participants were randomized for 40% to CG and 60% to IG. Randomization resulted in the following allocation: CG = 35 and IG = 51. We used separate blocks based on the intervention group to allocate participants to blood collection (CG = 15 and IG = 20). Due to the type of intervention, it was impossible to blind the participants and investigators to the intervention. It was not possible to blind users because of the differences in the interventions: while the usual care participated in collective activities, the IG had individual counseling. Also, one of the strategies used in the actions with the IG was the agreement of the treatment goals and clarification of the proposed objectives, which precluded its blindness. For all analyses, the participants were assigned to their original groups (IG or CG). The analyses were performed on the basis of intention to treat, which is described in greater detail in the statistical analysis section.

#### Intervention

Women within the CG maintained usual activities in the service, including physical exercise three times weekly and collective food and nutrition actions once per month. The nutrition actions include collective health education and workshops, lasting an average of 30 min. Women in the IG participated in the same activities as the CG, but they also received individual health counseling based on the TTM for weight management, performed once per month for 6 months.

The TTM was used with health education to foster a reflexive, proactive problem-solving, and participatory approach and to consider the different aspects of eating behavior and physical activity to promote the autonomous practice of healthy habits. Therefore, the participants were instigated to identify their obstacles to weight loss and constructed (together with the health professional) possible strategies to overcome them.

Prior to the intervention development, participants were classified into SOC using the Weight Loss Behavior-Stage of Change Scale regarding four habits related to obesity: portion control, dietary fat, fruit and vegetable intake, and physical activity. Intervention studies with multiple behaviors show greater compliance with body weight control than using a single behavior-based approach [5, 7, 10, 13]. Among the most evaluated behaviors are portion control, changes in dietary fat, fruit, and vegetable intake, and physical activity. These behaviors are key markers for weight loss [7].

To develop specific interventions, participants were classified into two groups for each habit: (i) pre-action, including women classified in the SOC (Table 1), pre-contemplation, contemplation, and preparation; and (ii) action, including participants who were classified in the action or maintenance stages [17]. The approaches were different for the pre-action and action groups for each assessed domain. In pre-action, the IG used processes of change predominantly based on cognitive changes to increase motivation and awareness about health challenges. The action (IG) group focused on processes of change related to behavioral changes, including more detailed guidance on nutrition concepts and physical activity [9]. The intervention was conducted to increase decisional balance and self-efficacy.

The intervention was conducted by a research team consisting of four dietitians. To standardize the intervention, the dietitians participated in periodic training on TTM theory and its application for weight control taught by a professor and researcher with experience of TTM. There were three face-to-face training sessions during the study period. In addition, nutritionists had support for possible questions throughout the study. The research team developed a manual to support the implementation of the intervention with modules for the four dimensions of TTM and each food habit and physical activity related to obesity.

The individual sessions generally lasted at least 30 min and focused on individualized goal setting, based on the patient’s SOC assessed at the baseline, and problem-solving. Verbal and written guidelines through simple language – according to individual participant needs – were used for guidance. Monthly visits assessed participant adherence to the agreed changes and identified the need for treatment revision. When a participant had difficulty following the treatment, discussions reinforced the importance of adherence and overcoming barriers, and new strategies were developed.

### Measures

Data were obtained from face-to-face interviews at two time points: (1) after randomization and before the beginning of the intervention; and (2) after 6 months of TTM intervention. The questionnaire included socio-demographic, economic (age, income, education, occupation), and health information (diabetes mellitus, arterial hypertension, self-health perception, body satisfaction, attempts to lose weight in the last month, physical exercise), dietary behaviors (number of daily meals, daily per capita sugar), and food intake (two 24-h dietary recalls), as well as measuring anthropometry (weight, height, waist circumferences - WC and hip circumferences). In addition, biochemical parameters [glucose, adiponectin, resistin serum levels] were obtained by blood tests. Inflammatory parameters were chosen for their relationship with obesity.

To analyze the food intake, the mean consumption from the two 24-h dietary recalls were calculated. The 24-h recall examined consumption over two distinct and nonconsecutive days, including weekends or holidays. A kit of homemade measures was provided to improve the estimation of the food quantities. Data obtained from 24-h recall were analyzed with Diet Win® Professional version 2.0, and the caloric and macronutrient intakes were classified according to gender and age recommendations [18, 19]. The subsample participants fasted when blood was collated for biochemical measurements. Blood collection was performed by nurses in the first and last week of the study, always in the morning, with the individual fasting for 12 h. For analysis of the biochemical measures, blood was collected and stored in a freezer at − 80 °C.

The version of the Weight Loss Behavior-Stage of Change Scale algorithm (validated [5], translated, back-translated, and adapted for use in Brazil) was used to identify the stages of change for weight management [20]. The instrument was also previously tested in PAS users to verify its applicability. The instrument assesses four habits related to weight management: (i) portion control, (ii) dietary fat intake, (iii) fruit and vegetable intake, and (iv) usual physical activity. For each habit, the participant evaluated their behavior according to statements ranging from 9 to 11 affirmatives. For each statement, the participant indicated the option that best described their behavior among the five available phrases, each referring to SOC, ranging from “I do not do this at least half the time now, and I have no plans to do this” (pre-contemplation) to “I do this at least half the time now and have been doing it regularly for more than 6 months” (maintenance). The food portion control evaluated the amount of food consumed during a meal and throughout the day, day-to-day eating control, and impulse to eat control into situations such as stress and depression. The dietary fat intake had information regarding consumption of chicken skin, meat with apparent fat, fat milk, fried foods, fast food, biscuits, butter or margarine, and sauces. The fruit and vegetable intake evaluated the portions consumed throughout the day, as well as their consumption in snacks or substitution for other food, such as sweets. The SOC for physical activity focused on evaluating domestic activities and active daily routines.

To identify the participant’s SOC for each of the evaluated habits, the option with the highest number of answers within the questions included in the evaluation was verified, and in case of a tie, the least advanced stage of behavior was considered.

At the 3- and 6-month follow-ups, the IG participants were evaluated for health counseling adherence, including identifying perceived barriers, benefits, and social support to make changes. This information was obtained through the participants’ self-report, who completed the follow-up.

The primary outcome was changed in weight from baseline to 6 months. Secondary outcomes included BMI, WC, energy consumption, glucose, adiponectin, and resistin. Other measures collected at baseline and 6 months were described. All data were collected by healthcare professionals who were properly trained and supervised by the researchers.

### Statistical analysis

The Shapiro-Wilk test was used to evaluate the distributions of the variables. Variables with normally distributed variables were presented as means and standard deviations (SDs), and asymmetric variables were presented as medians and interquartile ranges (P_25_ - P_75_).

The inter-group differences at the baseline were evaluated using the independent sample t-test, chi-square, and Fisher’s exact test for socioeconomic variables, health variables, and stages of change. Dieting habits, food intake, anthropometry, and biochemical parameters were not considered primary or secondary outcomes, but were evaluated in an intra-group comparison by the time of the follow-up using paired Student t, Wilcoxon, and McNemar tests. The test of homogeneity was performed for each of the primary and secondary outcomes. Analysis of covariance (ANCOVA) was used to assess intervention effectiveness to primary and secondary outcomes, adjusted for age, education, and baseline measurements. The adjustment measures were determined considering important factors associated with the outcomes according to a literature review and the groups’ differences at baseline. The results of the comparisons were corrected using the Bonferroni method.

Intention-to-treat analysis was performed to determine the effectiveness of the intervention and to keep all participants with non-missing baseline outcome measurements. In the case of missing values at 6-month (32.5%), imputation was used by replacing the baseline value following the Baseline Observation Carried Forward approach.

Statistical significance was attributed when *p*-values were less than 0.05. Analyses were performed using SPSS Statistics for Windows, version 17.0.

## Results

Of the 86 eligible women enrolled at the beginning of the study, 58 completed the 6-month intervention: 24 women in the CG (31.4% of attrition) and 34 in the IG (33.3% of attrition) (*p* = 0.85) (Fig. 1). In the CG, losses were related to non-attendance to the face-to-face interviews three consecutive times (*n* = 11). In the IG, losses were related to non-attendance three consecutive interviews (*n* = 6), as well as infrequent use of the service and without telephone contact (*n* = 3), those unable to continue the nutritional intervention because of personal problems (*n* = 6), and participants who refused to continue the nutritional intervention (*n* = 2). The CG participants who completed the trial had a lower per capita income than those that did not complete the trail [165.87 (86.67–240.00) and 345.87(180.00–244.00), respectively; *p* = 0.008]. After the intention to treat, an analysis verifies if the estimated data were similar to the collected information. The analysis showed the correlation for anthropometrics and eating habits with non-imputed data and imputed data (correlation = 1.000, *p* > 0.001 for all anthropometrics and eating habits).

At baseline, the CG and IG participants were approximately 55-years-old, and with a BMI above 30 kg/m^2^ (30.9–34.8). There were significant inter-group differences in education, self-health perception, and physical activity. Both groups reported low income per capita, low educational attainment, and did not perceive their health as well. Overall, most women were dissatisfied with their body shapes and had previous weight loss attempts. Most of the participants were classified in the action and maintenance SOC for all four aspects (Table 2).

**Table 2:** Baseline participant characteristics according to study group

Variables	Usual care control group (***n*** = 35)			Intervention Group (***n*** = 51)			***p*** value
	n	Values		n	Values		
**Socioeconomic variables**							
Age (years)	35	55	± 13.5	51	52.0	± 14.3	.25^1^
Monthly income per capita ($)	35	250.00	155.00–316.66	51	250.00	157.20–375.00	.61^2^
Education (years)	35	4.0	3.0–11.0	51	8.0	4.0–11.0	.07^2^
Occupation (%)							.28^3^
Fixed income	22	62.9	–	26	51.0	–	
Without fixed income	13	37.1	–	25	49.0	–	
**Health variables**							
Diabetes *mellitus* (%)	7	20.0	–	11	21.6		.88^3^
Arterial hypertension (%)	18	51.4		31	60.8		.41^3^
Self-health perceptions (%)							.02^3^
Very good/Good	24	68.6	–	22	43.1	–	
Moderate/Poor/Very poor	11	31.4	–	29	56.9	–	
Body satisfaction (%)							.12^4^
Satisfied	1	31.4	–	2	17.6	–	
Not satisfied	23	65.7	–	42	82.3	–	
Attempted to lose weight in the last 6 months (%)	25	71.4	–	41	80.4	–	.61^3^
Physical exercise (twice a week or more) (%)	34	97.1		49	96.1		0.79^3^
**Stages of change (%)**							
Portion control							.91^3^
Pre-action	10	28.6	–	14	27.4	–	
Action	25	71.4	–	37	72.5	–	
Dietary fat intake							0.57^4^
Pre-action	4	11.4	–	4	7.84	–	
Action	31	88.6	–	47	92.2	–	
Fruit and vegetable intake							.69^3^
Pre-action	11	31.4	–	14	27.4	–	
Action	24	68.6	–	37	72.5	–	
Physical activity							.01^3^
Pre-action	14	40.0	–	8	15.7	–	
Action	21	60.0	–	43	84.3	–	

^1^t student test, ^2^Mann-Whitney test, ^3^Chi-square test, ^4^Fisher’s exact test*.* Symmetric variables: mean ± standard deviation. Asymmetric variables: median (P_25_-P_75_)

After 3 and 6 months of intervention, approximately 97.0% of women in the IG reported having followed the intervention guidelines. The main perceived barriers to adherence were lack of time, financial difficulties, and lack of desire/motivation. Approximately 20% of the participants did not report adherence difficulties (Table 3).

**Table 3:** Follow-up adherence to guidelines, perceived barriers and benefits of participants in the intervention group

Variables (%)	Intervention Group (***n*** = 33^**a**^)				***p*** value^**1**^
	After three months		After six months		
	n	Frequency	n	Frequency	
**Guidelines put into practice**	32	97.0	32	97.1	1.000
Followed all guidelines	18	54.5	15	45.5	–
Followed the guidelines for some time, but then abandoned them	3	9.1	1	3.0	
Followed some of the guidelines	12	36.4	13	39.4	
Tried to follow the guidelines, but were unable	0	0.0	4	12.1	
**Barriers to guideline adherence**					
Lack of time	7	25.0	5	17.9	0.687
Financial difficulties	4	18.2	1	4.5	0.250
Lack of willingness/motivation	5	17.9	7	25.0	0.754
Without difficulties	8	25.0	7	21.9	1.000
**Benefit from nutritional health counseling**	30	93.8	31	96.9	1.000
Disposition	19	63.3	16	53.3	0.508
Weight reduction	9	30.0	9	30.0	1.000
Health improvement	5	26.3	6	31.6	1.000
Improved biochemical parameters	4	13.3	4	13.3	1.000
Improved intestinal functioning	6	20.0	6	20.0	1.000

Note: n refers to the actual number of responses to each item. Variables “Barriers to guideline adherence” and “Benefit from nutritional health counseling”: respondent could choose more than one answer options

^1^McNemar test. ^a^Data loss due to lack of information

Over 90% of participants who completed the 3- and 6-month follow-ups reported benefits associated with the health intervention (Table 3), which was confirmed by changes in eating habits and anthropometric and biochemical profiles observed at follow-up (Table 4).

**Table 4:** Change in control and intervention group after six months

Variables	Usual care control group (***n*** = 35)							Intervention group (***n*** = 51)						
	Baseline			After six months			***p*** value	Baseline			After six months			***p*** value
	n	Values		n	Values			n	Values		n	Values		
**Dieting behaviors**														
Number of daily meals	35	4.8	± .97	35	4.4	± 1.1	.008^1^	51	4.5	± 1.0	51	4.8	± 0.9	.03^1^
Daily per capita sugar (g)	35	33.3	8.3–66.7	35	41.7	13.9–66.7	.71^2^	51	28.3	12.5–55.5	51	25.0	0.0–41.7	.01^2^
Stage for portion control (%)							1.00^3^							.01^3^
Pre-action	10	28.6		9	25.7			14	27.4		7	13.7		
Action	25	71.4		26	74.3			37	72.5		44	86.3		
**Food intake**														
SFA (%)	35	7.7	5.6–10.5	35	10.1	7.4–13.7	0.001^2^	51	8.4	7.8–10.5	51	10.7	8.4–13.7	<.001^2^
MUFA (%)	35	8.3	6.1–10.3	35	10.3	7.2–13.5	.01^2^	51	8.3	7.5–9.3	51	9.4	7.8–3.2	.006^2^
**Anthropometry**														
Waist circumference (cm)	35	97.0	91.7–102.0	35	96.5	82.0–102.0	.81^2^	51	94.0	88.3–99.0	51	92.5	88.0–97.0	.01^2^
Weight (Kg)	35	80.2	± 12.3	35	81.1	± 11.5	.09^1^	51	80.4	± 11.9	51	79.9	± 11.6	.09^1^
BMI (kg/m^2^)	35	32.0	30.7–34.6	35	32.9	30.8–34.8	.05^2^	51	33.0	31.0–34.9	51	32.7	31.0–35.0	.11^2^
**Biochemical parameters**^**a**^														
Blood glucose (mg/dl)	35	92.5	86.7–101.0	35	90.1	79.0–101.0	.83^2^	51	85.6	81.4–90.7	51	82.7	72.6–87.0	.01^2^

Note: MUFA, monounsaturated fatty acids; SFA, saturated fatty acids. BMI, body mass index. ^1^paired Student t test; ^2^Wilcoxon test, ^3^McNemar test. ^a^Sub-sample. Symmetric variables: mean ± standard deviation; asymmetric: median (P_25_ - P_75_)

Analyses of the intervention effectiveness after adjusting for age, education, and baseline measurements revealed significant differences in body weight, BMI, and resistin between women in the IG and CG (Table 5). However, no difference was found in the secondary outcomes, including WC, energy consumption, and adiponectin.

**Table 5:** Comparison of the final adjusted means, according to groups allocation

Variables	Usual care control group					Intervention group					∆	CI (95%)	***p*** value ^**1**^
	n	MI	CI (95%)	FAM	CI (95%)	n	MI	CI (95%)	FAM	CI (95%)			
Weight (Kg)	35	80.2	76.0–84.4	81.2	80.3–82.0	51	80.4	77.0–83.7	79.8	79.1–80.5	−1.4	−2.5;-0.3	.01
BMI (kg/m^2^)	35	32.8	31.6–34.1	33.5	33.1–33.8	51	33.3	32.2–34.4	33.0	32.7–33.3	−0.5	−0.9; −0.5	.03
WC (cm)	35	96.8	94.4–99.3	95.0	93.5–96.5	51	93.3	90.1–96.4	93.7	92.6–94.9	−1.3	−3.2; 0.6	.19
Energy consumption (KJ)	35	1404.4	1254.2–1554.6	1465.4	1356.7	51	1513.2	1392.1–1634.3	1392.1	1302.3–1481.8	−73.4	− 216.0; 69.2	.31
Adiponectin^a^ (ng/mL)	15	29.7	20.1–39.3	24.9	19.0–30.7	20	23.9	16.4–31.4	25.1	20.0–30.1	0.2	−7.9;8.3	.96
Resistin^a^ (pg/mL)	15	4.8	2.1–7.5	3.8	2.4–5.2	20	4.8	3.6–6.0	5.8	4.6–7.0	2.0	−0.6; 3.9	.04
Blood glucose^a^ (mg/dl)	15	94.7	85.1–104.2	94.0	87.2–100.8	20	95.2	79.6–110.8	81.3	75.6–87.1	−12.6	−22.0;-3.3	.10

Note: *CI* confidence interval, *MI* mean initial, *FAM* final adjusted mean, *BMI* body mass index, *WC* waist circumference

^1^ANCOVA: adjusting for age, education, measured at baseline and followed the guidelines. ^a^Subsample. Δ = difference between final adjusted mean

## Discussion

The TTM-based intervention was shown to be an effective strategy for weight reduction in PHC, leading to positive effects concerning nutritional status, dietary behaviors, waist circumference, glucose, and resistin levels. The usual care within PAS was unsuccessful in controlling weight during the 6 months of the trial. These findings suggest the promise of the TTM-based intervention approach for weight-loss behaviors while indicating the need for more effective weight maintenance strategies for usual public health care and communities of low socioeconomic status.

To our knowledge, this is the first study in the literature that investigates the impact of an individualized multi-behavioral TTM intervention that addressed multiple behaviors for weight management on PHC, associated with inflammatory and metabolic evaluation. Furthermore, food and nutrient intake, eating habits, anthropometric characteristics, and biochemical and inflammatory profiles were simultaneously assessed.

The use of multi-behavioral interventions is a promising strategy to control multifactorial morbidities (such as overweight and obesity), especially when considering the complexity of behaviors associated with individual lifestyles [12]. Moreover, it should be considered as a maximized cost-effectiveness intervention, promoting action and paired actions, in which a change of one behavior can help to change another behavior [11].

To overcome impersonal program interventions, this study used an individualized intervention according to readiness for change that considers the complexity involved with food choices. Feasible strategies based on the economic, cultural, and social environments of participants, which enabled them to make healthy choices, were developed. At the 3- and 6-month follow-ups, the vast majority of participants reported that they followed guidelines. Adherence to treatment is one of the main determinants of its success and is influenced by multiple factors, such as recognition of risk behaviors and the development of self-care [21]. Also, participants perceived different health benefits with TTM-based intervention. This finding makes sense because TTM enables differentiated interventions according to individual subjective perception.

Despite following the guidelines, participants identified barriers to change that were addressed throughout the intervention. After 6 months, the main barrier identified was the motivation to follow weight loss treatment. This demonstrates the need for constant assessment of readiness for change to guide the intervention. In addition to specific guidelines to the SOC, strategies such as Social Cognitive Theory and Motivational Interviewing have proven effective for this type of obstacle [14].

Hence, while the CG presented unfavorable changes, the relatively high levels of adherence in IG were accompanied by beneficial changes in anthropometric measurements, diet habits, and biochemical parameters. These results suggest that the intervention increased their awareness of diet, but this must be confirmed through other studies. This probably encouraged participant autonomy and most likely led to changing parameters beyond those specifically addressed by the intervention [9, 22, 23].

Another important improvement presented by IG is the progression from early stages of change to final stages (action and maintenance) regarding portion control, which suggests increased food selectivity and impulse control, which in turn is an important indicator of the sustainability of behavior change [9, 22].

The SOC evolution and the eating habits changes that occurred in the IG might have helped the weight loss to occur. The differences between the IG and CG are relevant, especially considering the obesogenic environment of large cities and the positive effects that modest weight loss might have on reducing metabolic and cardiovascular risks [23]. Similar results in primary care were observed in a TTM-based weight-loss trial with a low-income sample, showing weight loss after 6 months [12]. Another trial using multi-behavioral TTM intervention also showed significant weight loss [13]. Some clinical trials that used different approaches, such as online and for telephone, reported weight loss ranging from 0.2 to 2.1 kg [7, 11, 24].

Despite being one of the target behaviors of the intervention, it is important to note that participants in both groups were not different in exercise practice throughout the trial (data not shown). The PAS routinely offers regular physical exercise, and all the participants were users of the service prior to the study. Therefore, everyone practiced physical activity, which is valuable for inflammation control, whether resulting from an isolated effect or as a precursor to reduced body weight [25]. The physical activity performed an average of 12 months prior to the study might have influenced the baseline levels of inflammatory mediators [25]; this reinforces the importance of this PHC service and multidisciplinary interventions for the promotion of health care.

Nevertheless, when comparing CG and IG, reduced levels of resistin were detected in the CG. The resistin is released from infiltrating white blood cells subsequent to subclinical chronic low-grade inflammatory response, accompanying obesity. Thus, the resistin level might be linked to the control of insulin resistance and metabolic syndrome [23]. The levels of resistin are related to other biochemical markers and can vary the concentration after intervention for weight loss. Other studies have reported either a reduction or increase in these concentrations, after intervention for weight loss, according to the characteristics of the participants [26, 27].

Some limitations should be highlighted. The high percentage of individuals in the final stages of change at baseline might have limited the effects of the intervention. Adherence to nutritional guidelines was not evaluated for participants who abandoned treatment. The difference between decisional balance and self-efficacy along the trial was not evaluated, but these constructs were considered for the development of the nutritional intervention. Since women from the CG and IG participated in the same usual activity, it is possible that some information leaked between the groups. Another limitation of this study was the difference in educational levels between participants in the CG and IG groups. However, all analyses were adjusted by education to minimize the impact of this variable on the results. Significant results of body weight change were not found in the intra-group analysis. However, although not significant, the results showed a tendency to reduce weight in the IG and gain in the CG, and this difference was significant in the inter-group comparison.

In 6 months, the attrition of both groups was 32.5%, reflecting the challenge of conducting an intervention study without offering payment to subjects. This might also reflect the PHC service, which presents a high turnover of users [28]. Intention-to-treat techniques were used to minimize the impact of loss. Also, the methods of this clinical trial did not allow blinding of the participants and professionals involved.

Another limitation was that the trial registration was performed after data collection. However, the study was submitted and approved to the Ethics Committee before recruiting participants, following all guidelines of the Declaration of Helsinki. The study was carried out and approved, taking into account the aspects recommended in a clinical trial. In addition, the data collection of this work was carried out in 2012. Therefore, differences in the context experienced today and in the time of data collection should be considered when interpreting the results. The study was conducted in a unit of the first implemented in the municipality. In addition, the high cost of biochemical analysis limited the expansion of the study to other units. There are currently 78 units in Belo Horizonte and 1811 throughout Brazil. However, it is important to note that after the study, PAS was expanded in Brazil and is today considered a useful space for the treatment of obesity and it is part of the network of attention to chronic diseases in the country [14]. Also, this study was performed in a PHC service in Brazil, and the results should not be generalized to populations with other characteristics. More studies are needed for other populations. Additionally, this study includes only the results of women. Few men were included in the PAS, which makes it impossible to obtain a sufficient sample to verify the effectiveness of the intervention. Studies using this population are necessary, considering its high prevalence of obesity and associated factors. Despite the limitations, this study showed that the TTM-based intervention for weight control in this scenario was effective and shows the relevance of these strategies in PHC for weight control.

## Conclusions

Individualized TTM-based interventions, when combined with usual care, might offer a viable and effective strategy for weight loss in primary health care and have a positive impact on inflammatory markers. The characteristics of the approaches used are in line with international guidelines aimed to prevent and control NCD by promoting interdisciplinary practices that are indispensable for the successful treatment of obesity and other NCD. Nevertheless, further research to identify additional strategies is needed to address barriers to weight maintenance among obese low-income women.

## Data Availability

The data sets in this study are available from the corresponding author on reasonable request.

## Abbreviations

NCD: Non-communicable diseases
PHC: Primary health care
TTM: Transtheoretical model
SOC: Stages of change
IG: Intervention group
CG: Comparison group
PAS: Programa Academia da Saúde
WC: Waist circumferences
SD: Standard deviations
BMI: Body mass index
